# 538. The Role of N-acetylcysteine on Post Covid-19 Pulmonary Fibrosis

**DOI:** 10.1093/ofid/ofab466.737

**Published:** 2021-12-04

**Authors:** Bernard Demot, Kristin Ivan Mark Hizon

**Affiliations:** Baguio General Hospital and Medical Center, Baguio City, Benguet, Philippines

## Abstract

**Background:**

Covid 19 have long lasting complications, from myalgia, body weakness to life debilitating strokes, and pulmonary fibrosis. Several mechanisms had been described but mostly viral or autoimmune which causes damages which leads to Acute respiratory distress syndrome. There is no approved treatment as of this time. Antifibrotic drugs use had been limited due to hepatoxicity, on top of Covid 19 hepatopathy. This study aims to describe the role of N-acetylcysteine on Post COVID 19 pulmonary fibrosis as an alternative treatment.

**Methods:**

Patients are admitted at Baguio General Hospital and Medical Center at the COVID wards. Patients are COVID confirmed by RT PCR nasopharyngeal swab. Patient who are classified as severe were given Dexamethasone, Enoxaparin and Remdesivir for 5-10 days. Patients who are not weaned off from O2 support underwent Chest CT scan. Patients with Extensive Fibrosis were then consented to undergo High Dose IV Infusion of N-acetylcysteine. (150mg/kg in 1st hour, 50mg/kg next 4 hours and 100mg/kg last 20 hours). Repeat Chest CT Scan was done.

**Results:**

Peripheral Bilateral Ground Glass Opacities and Pulmonary Consolidation was seen on pre-treatment CT Scans. Repeat CT scans showed significant regression of Ground Glass Opacities and Pulmonary Consolidation.

CT SCAN pre and post treatment

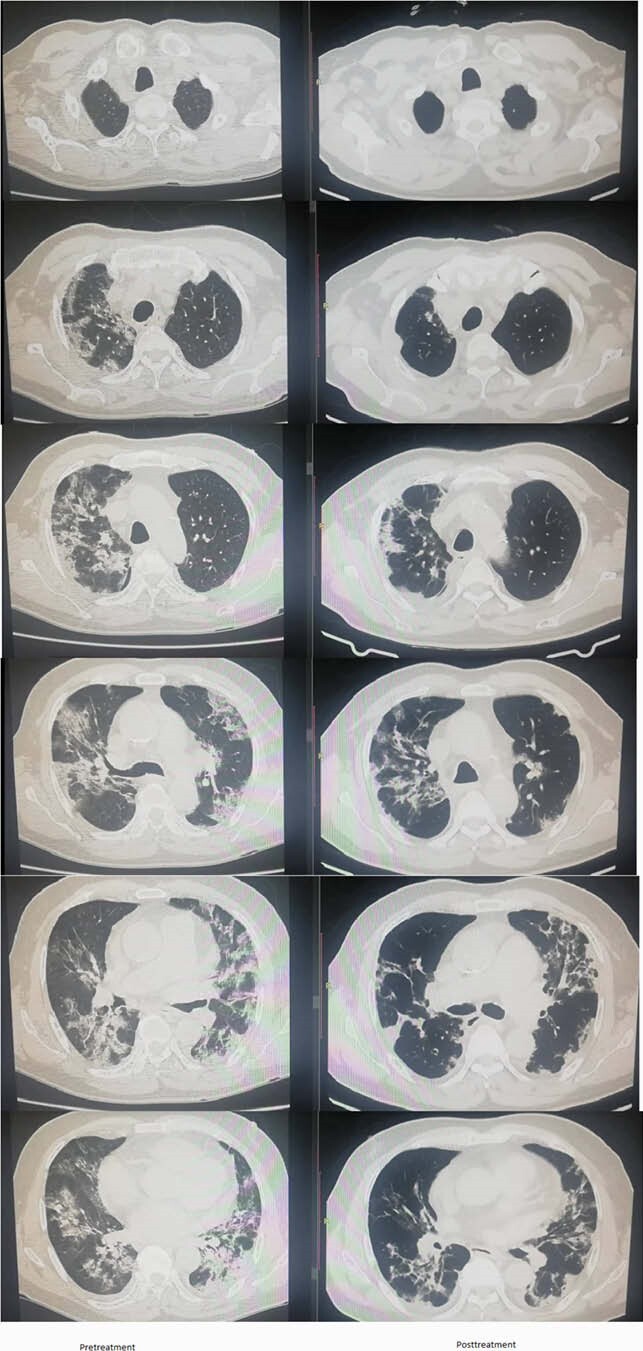

**Conclusion:**

High dose N-acetylcysteine showed promising results on Post COVID 19 Pulmonary Fibrosis.

**Disclosures:**

**All Authors**: No reported disclosures

